# Association of Pickleball Participation With Decreased Perceived Loneliness and Social Isolation: Results of a National Survey

**DOI:** 10.1177/21501319251385855

**Published:** 2025-10-21

**Authors:** Jordan D. Kurth, Jonathan Casper, Christopher N. Sciamanna, David E. Conroy, Matthew Silvis, Louise Hawkley, Madeline Sciamanna, Natalia Pierwola-Gawin, Brett R. Gordon, Alexa Troiano, Quinn Kavanaugh

**Affiliations:** 1 Pennsylvania State University College of Medicine, Hershey, USA; 2 North Carolina State University, Raleigh, USA; 3 Pennsylvania State University, University Park, USA; 4 The Bridge, NORC at the University of Chicago, IL, USA; 5 Queen’s University, Kingston, ON, Canada; 6 West Chester University, PA, USA; 7 Millersville University, PA, USA

**Keywords:** health promotion, physical activity, social determinants of health, primary care, lifestyle change

## Abstract

**Introduction/Objectives:**

One in four older adults in the United States (US) reports being socially isolated or lonely, leading to increases in the odds of having heart disease, a stroke, and dementia. Physical activity (PA) has many benefits in this population, both physical and psychological. One such type of PA growing in popularity is pickleball. Our objective was to understand the relationship of pickleball participation with perceived loneliness and social isolation among older adults in the US.

**Methods:**

We conducted a national cross-sectional survey of 825 US adults over age 50 years. We compared the effect of 3 levels of pickleball play history (never played (NP); played previously, not currently (PP); and currently play (CP)) on loneliness and social isolation.

**Results:**

Most participants (65%) who had ever played pickleball were still currently playing. Over half of the sample (57%) reported being lonely. The adjusted odds of being lonely were greater for NP (adjusted odds ratio 95% CI = 1.53, 1.04-2.23), and PP (1.95, 1.24-3.05) groups. Compared to NP, PP, and CP groups were found to be associated with having made more social connections and reporting a greater number of social engagements, thus appearing to experience less social isolation.

**Conclusion:**

Older adults who participate in pickleball had an associated decreased perceived loneliness and reduced risk of social isolation. Further research is needed to determine if recommendations for those able to physically participate in pickleball might result in similar changes to loneliness and social isolation.

## Introduction

Social isolation and loneliness are among the most common, yet untreated, problems, affecting 1 in 4 older adults.^1,2^ National surveys in the United States (US) report that between 24 to 41% of older adults report feeling socially isolated or lonely.^2–4^ Furthermore, studies have found that poor social relationships, assessed as social isolation or loneliness, increase risk of heart disease by 29%, stroke by 32%, and dementia by 50% among older adults.^2^ As part of a self-perpetuating cycle, decreased physical function and declines in physical activity (that may be driven by social isolation in the first place) are also associated with social isolation.^5^ Although systematic reviews of interventions to reduce social isolation show moderate effects, the interventions tested (eg, discussion groups and one-on-one counseling) have been resource intensive and not easily scaled.^6–8^ Systematic reviews focused on individual- and group-level physical activities aimed at reducing social isolation identified no significant effects;^8^ however, those physical activity interventions were not designed specifically to increase opportunities for social contact.

Older adults report preferring social opportunities based on interest.^9^ One potential social opportunity is pickleball. Pickleball is the fastest growing sport in the US and may be particularly well suited to older adults because it has reduced barriers to entry in terms of capability, opportunity, and motivation. There were 8.9 million people who played pickleball in the US in 2022, growing 156% over the previous 3 years. Over the next 5 years, it is expected that 25 000 courts will be built – an investment in $900 million in local infrastructure.^10^ Players aged 55 years and up make up the largest share of pickleball players (19.8%) and many play after age 70 years, making it an ideal intervention for older adults.^10^ Pickleball gear is inexpensive and courts are widespread, making it potentially far more cost effective than other loneliness interventions. The reduced size of the court (requiring less running) and the flatness of the paddle rather than a racket (which makes ball control easier) may improve longer-term participation since self-efficacy is one of the strongest predictors of physical activity uptake and maintenance.^11^ Pickleball is highly social, as most games are doubles (4 players at a time) and is easier to play while socializing due to the smaller court (30% of the size of a tennis court). The culture of pickleball is also known for its good sportsmanship and welcoming culture.^12^

A recent systematic review observed that pickleball play was associated with significant improvements in mental and social health, including demonstrated associations with well-being, decreased incidence of depression and improved subjective well-being and life satisfaction.^13–15^ However, pickleball’s association with perceived loneliness and social isolation have not yet been studied. In this study, we set out to understand the relationship between pickleball play and loneliness and social isolation among older adults in the United States. We hypothesized that older adults who play pickleball would report less perceived loneliness and social isolation. Our hypotheses were based on the premise that pickleball has the potential to reduce loneliness by improving the quality of relationships and to reduce social isolation by increasing the number of social connections and engagements.

## Methods

This institutional review board exempted study (IRB# STUDY00022738) used an anonymous online cross-sectional survey to understand differences between US adults over 50 who have never played pickleball (NP group), have previously played (PP group), and who currently play(CP group). Throughout this manuscript, the reporting of data are in-line with the Strengthening the Reporting of Observational Studies in Epidemiology (STROBE) guidelines for observational cross-sectional studies.^16^ The survey was conducted via a survey panel arranged using a commercial survey company (Qualtrics). Qualtrics uses a network of consumers who have opted-in to participate in research from many suppliers, including advertisements and promotions on smartphones, referrals from membership lists, social networks, mobile games, banner advertisements, mail-based recruitment campaigns, and others.^17,18^ To ensure data quality, surveys included (1) attention checks (ie, factual questions with correct answers) and (2) speeding checks (ie, eliminating responses from those who completed the online survey in less than one-third the median duration of survey completion). Qualtrics was compensated for facilitating the collection of this data at a cost of approximately $20 per participant. These methods have been used previously for large-scale, targeted survey research.^19^

### Participants

Participants were required to be at least 50 years of age, located in the United States, and fluent in the English language. Additional quotas were placed on recruitment (50/50 split, both males/females and having ever played pickleball/never having played) to ensure a sample appropriate for the planned analyses. Data collection occurred during July of 2023.

### Instruments and Measures

#### Demographic Information and Covariates

Demographic and anthropometric characteristics, such as age, gender, race, ethnicity, height, and weight were assessed, and body mass index (BMI) was calculated using the standard formula.^19^ Smoking status was assessed using a single item adapted from the Behavioral Risk Factor Surveillance System (BRFSS), “*Do you use a tobacco product every day?*”.^20^ Medical history was assessed using questions (also from BRFSS) assessing presence or absence of: diabetes, high cholesterol, heart disease, osteoporosis, hypertension, stroke, arthritis, assistive device use, and difficulty walking or climbing stairs.^20^ Anxiety and depressive symptoms were measured by the short forms of the Patient-Reported Outcomes Measurement Information System (PROMIS) Anxiety and Depressive Symptoms scales were used.^21^

#### Physical Activity (PA)

Seven-day recall of physical activity was measured using the International Physical Activity Questionnaire – Short Form (IPAQ-SF).^22^ Participants reported the frequency and duration of 3 different intensities of physical activity (walking, moderate, and vigorous) and 1 additional modality (strength training) over the past week. Consistent with IPAQ scoring protocol, outlying data points were defined as those of 960 min or more of activity on any 1 day^23^ and removed. Moderate-to-vigorous physical activity (MVPA) was calculated by the sum of the products of frequency and duration reported for moderate PA and vigorous PA, respectively.

#### Pickleball Play History

Participants fell into 1 of 3 categories describing their pickleball play history. First, participants were asked if they have ever played pickleball; those responding “No” to this question were categorized as never having played pickleball (NP group). Those that answered “Yes” to this question then responded to a question asking if they still currently play pickleball. Those that responded “No” to this question were categorized as having previously played but not currently playing (PP group); those responding “Yes” were categorized as currently playing pickleball (CP group).

#### Loneliness

Loneliness was measured using the 3-Item Loneliness Scale, a validated, reliable, self-administered questionnaire that measures how often participants feel left out, feel isolated, or that they lack companionship.^24^ For each item, participants were asked if they feel that way “hardly ever,” “some of the time,” or “often” (assigned values 1, 2, and 3, respectively). Responses are summed to create a total loneliness score that ranges from 3 to 9, with higher values representing more frequent loneliness. Our primary analysis evaluated loneliness as a dichotomous outcome. For this analysis, we classified participants as “lonely” if they responded “some of the time” or “often” to any of the 3 components. We classified subjects as “not lonely” if they responded “hardly ever” to all 3 components. These cut-points for loneliness have been shown to predict increased risk of functional decline and death.^25^ To test the sensitivity of these cut-points, we also performed sensitivity analyses using alternative definitions of the loneliness outcome.^25^

#### Social Isolation (Social Connections and Engagement Variables)

All participants responded yes or no to the question (emphasis included in survey), “*Have you made any social connections (or friends) based on your participation in* ***any type of exercise/physical activity****?*”. Those participants who responded yes were asked, “*Approximately how many social connections (or friends) have you made based on that* ***exercise/physical activity participation****?*”. Next, participants were asked if they had socialized with these friends outside of physical activity (PA) participation, and if so, asked, “*How many times per month do you socialize with friends that you have made through* ***exercise/physical activity participation****?*”. Only participants who had reported playing pickleball previously were asked the same questions as above, specific to their pickleball participation. The emphasized portions of the questions above were changed to “***pickleball participation only***”.

#### Analysis

All analyses were conducted using Stata BE version 17 (StatCorpLP, College Station, TX). Participants were grouped according to their pickleball playing history. Descriptive statistics (mean (SD) or n (%)) were calculated for demographic and physical activity variables. One-way analysis of variance (ANOVA) tests were used to identify differences in demographics. A multivariate logistic regression (OR, 95% CI) was performed to evaluate the impact of the 3 pickleball playing history groups on the adjusted odds (AO) of being lonely, having made social connections from PA, and having made social connections from pickleball. To account for the impact of physical health on social well-being,^26^ all odds ratios were adjusted for age, sex, MVPA, smoking, diabetes, high cholesterol, heart disease, osteoporosis, hypertension, stroke, arthritis, assistive device use, and walking difficulty.

Additionally, 2 analyses of covariance (ANCOVA) were conducted on number of social connections made from PA and the number of non-PA engagements per month. These models focused on evaluating differences between those that had never played pickleball, those that had played previously but did not currently, and those currently playing. Similarly, to account for the impact of physical health on social well-being ^26^, each model controlled for age, sex, MVPA, smoking, diabetes, high cholesterol, heart disease, osteoporosis, hypertension, stroke, arthritis, assistive device use, and walking difficulty. Additional comparisons for the number of social connections made from pickleball and the number of non-pickleball engagements per month were also conducted between those that had played previously but did not currently and those currently playing. These results are reported as estimated marginal means (EMMs). Eta squared values (η^2^) were calculated to assess the magnitude of group differences using the following cut-points for all ANCOVAs: η^2^≥.01 small, η^2^≥.06 medium, and η^2^≥.14 large effect.^27^

## Results

### Participant Profile

A total of 960 participants consented to be screened for eligibility. Participants that were not at least 50 years old (n = 65), that failed the attention check (n = 4), and that did not complete the survey (n = 66) were removed. Therefore, the final sample size included 825 participants. By study design, approximately half of participants were female. The mean participant age was 61 years and the majority (89%) reported their race/ethnicity to be non-Hispanic white. More than 40% had attended at least 4 years of college. A demographic summary is provided in Table 1.

**Table 1.** Participant Description.

		Pickleball history		
Variable	Overall (N = 825) (SD)	NP (n = 415) (SD)	PP (n = 144) (SD)	CP (n = 266) (SD)
Age, years	60.5 (7.7)	62.8 (8.4)^a^	57.6 (5.8)^b^	58.5 (6.2)^b^
Gender, female	406 (49.2%)	108 (26.0%)^a^	107 (74.3%)^b^	191 (71.8%)^b^
Race, White	734 (89.0%)	372 (89.6%)	132 (91.7%)	230 (86.5%)
Education, 4-year college	358 (43.4%)	145 (35.0%)^a^	58 (40.1%)^b^	155 (58.3%)^c^
Medical History				
Overweight BMI >24.9, n	615 (74.6%)	338 (81.5%)^a^	101 (70.1%)^b^	176 (66.2%)^b^
Diabetes, n	166 (20.1%)	94 (22.7%)	30 (20.8%)	42 (15.8%)
High Cholesterol, n	386 (46.8%)	207 (49.9%)	68 (47.2%)	111 (41.7%)
Heart disease, n	63 (7.6%)	42 (10.1%)^a^	9 (6.3%)^b^	12 (4.5%)^b^
Osteoporosis, n	88 (10.7%)	35 (8.4%)	21 (14.6%)	32 (12.0%)
Hypertension, n	410 (49.7%)	238 (57.4%)^a^	68 (47.2%)^a^	104 (39.1%)^b^
Arthritis, n	328 (39.8%)	175 (42.2%)	63 (43.8%)	90 (33.8%)
Stroke, n	33 (4.0%)	21 (5.1%)	5 (3.5%)	7 (2.6%)
Co-Morbidities (of 8 above)	2.5 (1.5)	2.8 (1.5)^a^	2.5 (1.5)^a^	2.2 (1.5)^b^
Physical Function				
Mobility device use, n	91 (11.0%)	62 (14.9%)^a^	14 (9.7%)^a^	15 (5.6%)^b^
Fell in past year, n	296 (35.9%)	156 (37.9%)	59 (41.6%)	81 (30.6%)
Serious difficulty walking or climbing stairs, n	148 (18.2%)	94 (22.7%)^a^	29 (20.1%)^a^	25 (9.4%)^b^
PA over past 7 days				
Moderate PA, min	161.6 (189.2)	130.3 (177.8)^a^	167.1 (186.4)^a^	207.9 (198.9)^b^
Vigorous PA, min	73.0 (133.0)	54.0 (122.5)^a^	78.4 (131.7)^a^	99.5 (144.6)^b^
Lonely, n	467 (56.6%)	235 (56.6%)^a^	97 (67.4%)^a^	135 (50.8%)^b^
Made friends from PA, n	508 (45.8%)	190 (45.8%)^a^	106 (73.6%)^b^	212 (79.7%)^b^
Number of connections from any PA	5.6 (13.0)	4.3 (12.9)^a^	7.7 (16.1)^b^	6.3 (11.1)^a^
Non-PA engagements per month with connections from PA any	1.8 (5.1)	1.2 (3.6)^a^	2.7 (9.5)^b^	2.2 (3.6)^b^

*Note.* Values with different superscript letters (^a^, ^b^, and ^c^) represent Bonferroni-corrected *P* < .05.

Abbreviations: BMI, body mass index; PA, physical activity.

Quota sampling resulted in a near equal split of participants having played pickleball previously (n = 410) compared to those that had never played pickleball (n = 415). Of those participants who had ever played pickleball, 65% were still playing (n = 266/410). Over half of the entire sample (56.6%) reported being lonely.

### Loneliness Differences Based on Pickleball Play History

**Figure 1.**
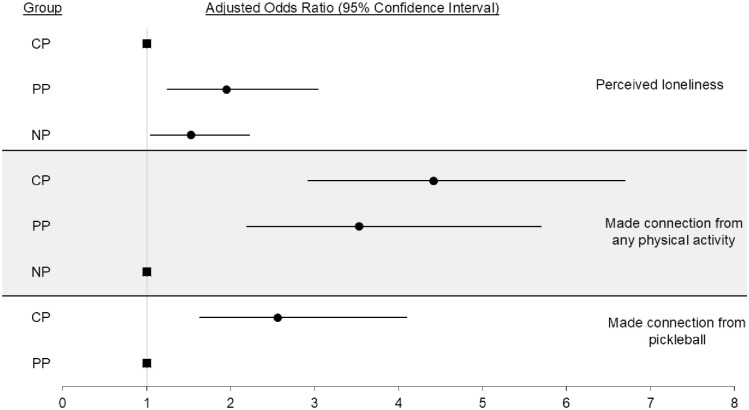
Adjusted odds ratios for perceived loneliness, connections made from any type of physical activity, and connections made from pickleball.

There was a significant difference in the adjusted odds (AO) of being lonely based on pickleball play history. AO of being lonely (OR, 95% CI) were greater for both the NP (1.53, 1.04-2.23, *P* = .03), and PP groups (1.95, 1.24-3.05, *P* < .01) compared to the CP group (referent; see Figure 1). The results of additional sensitivity analyses conducted were consistent with the above results (Supplemental Table 1).

### Social Connections (From Any PA) Differences Based on Pickleball Play History

The AO of making of social connections from any type of PA based on pickleball play history were greater (OR, 95% CI) both the PP (3.53, 2.19-5.70, *P* < .01) and CP groups (4.42, 2.92-6.72, *P* < .01) compared to those in the NP group (referent; see Figure 1). There was also a significant difference in the number of social connections made from any type of PA after controlling for covariates based on pickleball play history (partial η^2^ = .02). On average (EMM), participants in the NP group had made 3.8 social connections from any type of PA, PP group had made an average of 8.7 social connections from any type of PA, and the CP group had made an average of 6.7 social connections from any type of PA. There was also a significant difference in the number of social engagements per month with social connections made from any type of PA, after controlling for covariates, based on pickleball play history (partial η^2^ = .01). On average, the NP group had 1.2 engagements per month outside of PA, while the PP group had an average of 2.8 engagements (*P* < .01). There was no significant difference between the NP group and the CP group (1.2 vs 2.2; *P* > .05).

### Social Connections (From Pickleball) Differences Based on Pickleball Play History

The AO of having made social connections from pickleball based on pickleball play history (OR, 95% CI) were greater the CP group (2.56, 1.63-4.01, *P* < .01) compared to the PP group (referent; see Figure 1). There was also a significant difference in the number of social connections made from pickleball, after controlling for covariates, based on pickleball play history (partial η^2^ = .16). On average (EMM), the PP group had made an average of 1.6 social connections from pickleball and the CP had made an average of 3.6 social connections from pickleball (*P* < .01). There was no significant difference in the number of social engagements per month with social connections made from pickleball, after controlling for covariates, based on pickleball play history (partial η^2^ = .03). On average, the PP group had an average of 1.5 engagements while the CP had an average of 1.7 engagements (*P* > .05).

## Discussion

Our hypothesis was that pickleball playing would positively impact perceived loneliness and social isolation among older adults. The results demonstrate that after adjusting for demographic variables, current pickleball players reported lower odds of being lonely than those who either never played in the past or played in the past but do not currently play. Furthermore, individuals who had played pickleball had significantly higher odds of having made social connections from PA and had more non-PA social engagements. Participants who had played pickleball either currently or in the past were more than 3 times as likely as those who had never played to have made a social connection from any type of PA.

While the study was conducted with a national sample in a commonly used survey panel, the conclusions must be judged with several limitations in mind. First, the sample was of individuals with Internet access, yet only 75% of older adults report having access to the Internet.^28^ A nationwide survey conducted in 2020 using the same Qualtrics panel methodology of the current study observed greater rates of college completion (55.8%) than observed by the Census Bureau (37.5%),^29^ suggesting that the sampling method used by Qualtrics may be biased toward respondents that are more educated than the general public ^28^. This, in addition to imposing a quote that 50% of our sample had previously played pickleball, may be related to why the rates of serious difficulty walking or climbing stairs in this study (18%) were lower than the percentage of adults 65 years and older reporting difficulty “walking or climbing stairs” (26.9%) in a national study in 2016.^30^ Additionally, the survey was unable to capture the full complexities of social networks. The extent to which the increase in social connections contributes to the decrease in loneliness is a question for future research. Relatedly, extraversion is known to be associated with greater physical activity,^31^ and may be a contributing factor to both pickleball participation and wellness outcomes. Finally, as a retrospective cross-sectional study, it is not possible to determine causation, but only the association of the relationship between pickleball and perceived loneliness and social isolation. It is possible that other variables not measured impact the outcomes based on the history of playing pickleball. A prospective, randomized controlled trial could help determine the impact of playing pickleball on perceived loneliness and social isolation, along with other behavioral health outcomes. Further research to explore these potential impacts is indicated.

## Conclusion

Results from a large, national, cross-sectional study demonstrate that older adults who played pickleball were associated with a range of positive outcomes, including less perceived loneliness and less social isolation. This study adjusted for several demographic characteristics (eg, chronic conditions, age, gender, and BMI) and compared pickleball players to other non-participating adults, which strengthens these conclusions. These positive outcomes, coupled with the organic growth of the sport, suggest that a future prospective experimental study of pickleball playing may be warranted to define the impact of pickleball on the lives of older adults in the US.

## Supplemental Material

sj-docx-1-jpc-10.1177_21501319251385855 – Supplemental material for Association of Pickleball Participation With Decreased Perceived Loneliness and Social Isolation: Results of a National Survey Supplemental material, sj-docx-1-jpc-10.1177_21501319251385855 for Association of Pickleball Participation With Decreased Perceived Loneliness and Social Isolation: Results of a National Survey by Jordan D. Kurth, Jonathan Casper, Christopher N. Sciamanna, David E. Conroy, Matthew Silvis, Louise Hawkley, Madeline Sciamanna, Natalia Pierwola-Gawin, Brett R. Gordon, Alexa Troiano and Quinn Kavanaugh in Journal of Primary Care & Community Health

sj-docx-2-jpc-10.1177_21501319251385855 – Supplemental material for Association of Pickleball Participation With Decreased Perceived Loneliness and Social Isolation: Results of a National Survey Supplemental material, sj-docx-2-jpc-10.1177_21501319251385855 for Association of Pickleball Participation With Decreased Perceived Loneliness and Social Isolation: Results of a National Survey by Jordan D. Kurth, Jonathan Casper, Christopher N. Sciamanna, David E. Conroy, Matthew Silvis, Louise Hawkley, Madeline Sciamanna, Natalia Pierwola-Gawin, Brett R. Gordon, Alexa Troiano and Quinn Kavanaugh in Journal of Primary Care & Community Health
