# Supplementary material for: Association of Pickleball Participation With Decreased Perceived Loneliness and Social Isolation: Results of a National Survey
Source: J Prim Care Community Health. 2025 Oct 21;16:21501319251385855. doi: 10.1177/21501319251385855 (PMC12553873; doi:10.1177/21501319251385855)
Supplement: sj-docx-1-jpc-10.1177_21501319251385855 – Supplemental material for Association of Pickleball Participation With Decreased Perceived Loneliness and Social Isolation: Results of a National Survey [file sj-docx-1-jpc-10.1177_21501319251385855.docx]

Supplement Table 1. Sensitivity Analyses

|  | Definition of Loneliness | | | | | |
| --- | --- | --- | --- | --- | --- | --- |
| Pickleball Play History | Dichotomized | At least one response | | More than one response | | Continuous |
|  |  | Moderate | Severe | Moderate | Severe |  |
| NP | 1.53^b^ (1.04-2.23) | 1.12  (0.78-1.63) | 3.75^a^ (2.14-6.59) | 0.61^a^ (0.40-0.93) | 2.66^a^ (1.74-4.09) | 4.88^a^ (4.69-5.07) |
| PP | 1.95^a^  (1.24-3.05) | 1.49  (0.97-2.28) | 2.82^a^ (1.54-5.17) | 1.36 ^b^ (0.86-2.13) | 1.60^b^ (0.99-2.58) | 4.70^a^ (4.40-5.01) |
| CP | Referent^b^ | Referent | Referent^b^ | Referent^b^ | Referent^b^ | 4.21^b^  (3.98-4.44) |

*Note.* Values with different superscript letters (^a^ or ^b^) represent Bonferroni-corrected *p* < 0.05.

Sensitivity analyses were performed to compare the patterns of significance between the dichotomized outcome of loneliness with three alternative operationalizations of the loneliness outcome (at least one response, more than one response, continuous). For the first comparison, we classified the participants as “moderately lonely” if they responded “some of the time” to any component, and “severely lonely” if they responded “often” to any component. For the next comparison, participants were classified as “moderately lonely” if they responded at least “some of the time” to one component only, and they were classified as “severely lonely” if the responded at least “some of the time” to two or three components. For the last comparison, we analyzed the items as a continuous scale, giving one point for each component answered some of the time and two points for each item answered often.
